# Automated Integration of AI Results into Radiology Reports Using Common Data Elements

**DOI:** 10.1007/s10278-025-01414-9

**Published:** 2025-01-27

**Authors:** Garv Mehdiratta, Jeffrey T. Duda, Ameena Elahi, Arijitt Borthakur, Neil Chatterjee, James Gee, Hersh Sagreiya, Walter R. T. Witschey, Charles E. Kahn

**Affiliations:** 1 Department of Radiology, University of Pennsylvania Perelman School of Medicine, 3400 Spruce St., Philadelphia, PA 19104 USA; 2 Information Services, University of Pennsylvania Health System, Philadelphia, PA USA; 3 Leonard Davis Institute of Health Economics, University of Pennsylvania, Philadelphia, PA USA; 4 Present Address: Department of Radiology, Northwestern University, Chicago, IL USA; 5 Institute for Biomedical Informatics, University of Pennsylvania, Philadelphia, PA USA

**Keywords:** Radiology, Reporting, Common data elements, Standards, Artificial intelligence, Interoperability

## Abstract

Integration of artificial intelligence (AI) into radiology practice can create opportunities to improve diagnostic accuracy, workflow efficiency, and patient outcomes. Integration demands the ability to seamlessly incorporate AI-derived measurements into radiology reports. Common data elements (CDEs) define standardized, interoperable units of information. This article describes the application of CDEs as a standardized framework to embed AI-derived results into radiology reports. The authors defined a set of CDEs for measurements of the volume and attenuation of the liver and spleen. An AI system segmented the liver and spleen on non-contrast CT images of the abdomen and pelvis, and it recorded their measurements as CDEs using the Digital Imaging and Communications in Medicine Structured Reporting (DICOM-SR) framework to express the corresponding labels and values. The AI system successfully segmented the liver and spleen in non-contrast CT images and generated measurements of organ volume and attenuation. Automated systems extracted corresponding CDE labels and values from the AI-generated data, incorporated CDE values into the radiology report, and transmitted the generated image series to the Picture Archiving and Communication System (PACS) for storage and display. This study demonstrates the use of radiology CDEs in clinical practice to record and transfer AI-generated data. This approach can improve communication among radiologists and referring providers, harmonize data to enable large-scale research efforts, and enhance the performance of decision support systems. CDEs ensure consistency, interoperability, and clarity in reporting AI findings across diverse healthcare systems.

## Introduction

The application of artificial intelligence (AI) in radiology has opened new avenues to enhance diagnostic accuracy, streamline radiology workflow, and improve patient care. AI algorithms are increasingly able to perform complex tasks: to detect abnormalities, segment anatomical structures, and quantify pathological features. As of August 2024, the U.S. Food and Drug Administration has authorized more than 720 AI-enabled medical devices for use in radiology, yet there have been limited attempts to standardize reporting [1, 2]. For these advances to improve patient care, it is essential that the output from AI models be integrated easily and effectively into the key work product of diagnostic radiology, namely the radiology report.

Common data elements (CDEs) are units of information that consist of a specific, precise question and the corresponding set of allowable answers [3]. The use of a predefined value set is a fundamental aspect of CDEs—one that allows them to facilitate the uniform collection and exchange of data, ensuring consistency and interoperability across diverse healthcare systems [4]. In the context of medical research and clinical practice, the use of CDEs allows for data to be comparable and interoperable, which is essential for high-quality, reproducible research. CDEs are data that are “collected and stored uniformly across institutions and studies and are defined in a data dictionary” [5].

In radiology, CDEs provide a standardized approach to collect and report information extracted from radiological examinations; they can define patient demographics, imaging parameters, measurements, qualitative assessments, and diagnostic interpretations. By providing a clear framework for documentation, CDEs help maintain consistency and enable efficient data analysis, thereby supporting evidence-based clinical decision-making and advancements in patient care. Development, curation, and translation of CDEs into clinical practice has been a key informatics initiative in radiology [6]. A collaborative effort of the American College of Radiology (ACR) and the Radiological Society of North America (RSNA) has produced the *RadElement.org* resource, which offers both a web-based viewer and an application programming interface (API) for automated queries [7]. New CDEs can be submitted to *RadElement.org*; detailed authoring instructions are available [8]. For each CDE, authors should identify the specific clinical need that the CDE will address, select a descriptive and concise name, and specify the appropriate data type (such as integer, numeric, Boolean, or value set) corresponding to the imaging property or finding being represented.

The development of CDEs involves a rigorous process that includes consensus-building among experts in relevant fields. This process ensures that each element is clearly defined, clinically relevant, and widely acceptable. For radiology reports specifically, CDEs have been pivotal in advancing structured reporting—a format which enhances report clarity by systematically organizing information according to these predefined elements. Structured reporting using CDEs not only improves the quality of communication between radiologists and other healthcare providers but also facilitates automated extraction of data for research purposes without the loss of meaning or context. This article describes an innovative approach that utilizes CDEs to embed measurements derived from AI models directly into radiology reports in clinical practice.

## Methods

**Fig. 1:**
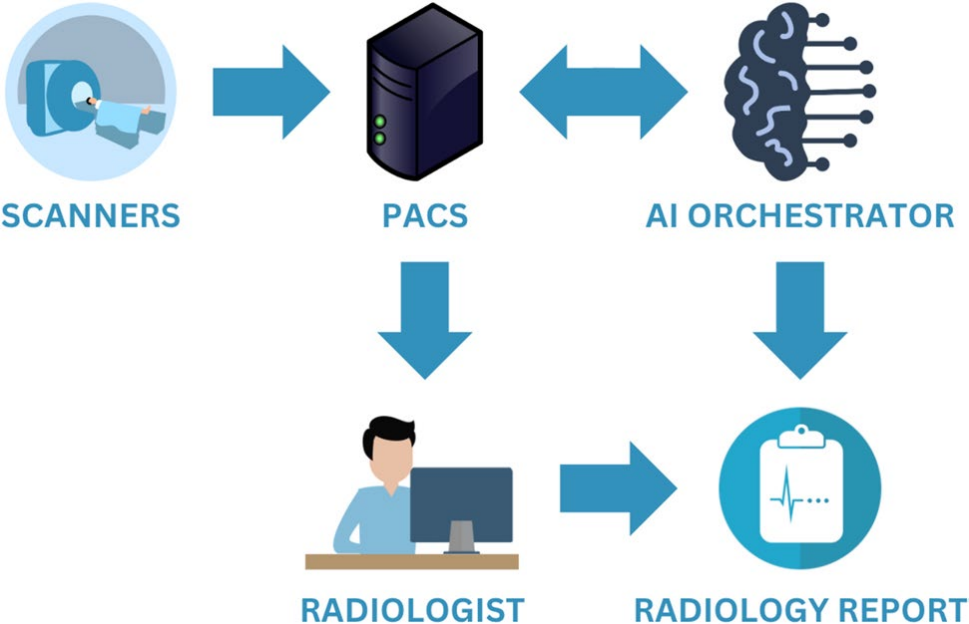
Flow diagram for AInSights, a machine learning-based platform for real-time clinical image analysis. AInSights uses deep learning algorithms to analyze uploaded images and generate clinical predictions, which are efficiently integrated into radiology reports with the assistance of an AI orchestrator

The AInSights platform, developed at the University of Pennsylvania Health System, includes a deep learning-based image analysis system and an AI “orchestrator” to monitor and coordinate the exchange of information between clinical and AI systems [9]. The platform has been implemented within a large, multi-hospital academic health system to provide real-time analysis of patient-care data and to support research applications (Fig. 1). The system applies a deep learning convolutional neural network for anatomic segmentation of CT; its initial clinical application has been volumetry of liver and spleen and opportunistic detection of hepatic steatosis in patients undergoing non-contrast CT examination of the abdomen and pelvis [10]. The system applied criteria for hepatic steatosis as described by Hamer et al. [11].

To capture the measurements generated by AInSights, the investigators defined a set of CDEs (Table 1). The proposed CDEs were submitted for consideration to the ACR-RSNA CDE initiative, where each data element was assigned a unique identifier, and its characteristics were defined. After review and minor revisions, the CDEs were published in March 2023 as CDE Set 174, “Morphometric CT Quantification of Liver, Spleen, and Abdominal Fat” (https://radelement.org/home/sets/set/RDES174).

**Table 1:** Identifier (ID), name, and unit of measurement of selected elements of CDE Set 174, “Morphometric CT Quantification of Liver, Spleen, and Abdominal Fat.” ***HU*** Hounsfield units

ID	Name	Unit
RDE1193	Liver-spleen CT attenuation difference	HU
RDE1194	Liver noncontrast CT attenuation mean	HU
RDE1195	Liver noncontrast CT attenuation median	HU
RDE1196	Liver noncontrast CT attenuation maximum	HU
RDE1197	Liver noncontrast CT attenuation minimum	HU
RDE1207	Spleen noncontrast CT attenuation mean	HU
RDE1208	Spleen noncontrast CT attenuation median	HU
RDE1209	Spleen noncontrast CT attenuation maximum	HU
RDE1210	Spleen noncontrast CT attenuation minimum	HU
RDE1220	Liver volume	mL
RDE1221	Spleen volume	mL

The AInSights system applied two integration profiles defined for trial implementation by the Integrating the Healthcare Enterprise (IHE) initiative: AI Workflow for Imaging (AIW-I) [12] and AI Results (AIR) [13]. AIW-I is a widely applicable framework for AI inference tasks on imaging data that defines steps for image acquisition, submission of an AI service request, AI-based image processing and analysis, and distribution of the image analysis results to the Picture Archiving and Communication System (PACS). The AIR profile establishes a standard for the storage, retrieval, and display of results from analyses conducted upon medical imaging data.

**Fig. 2:**
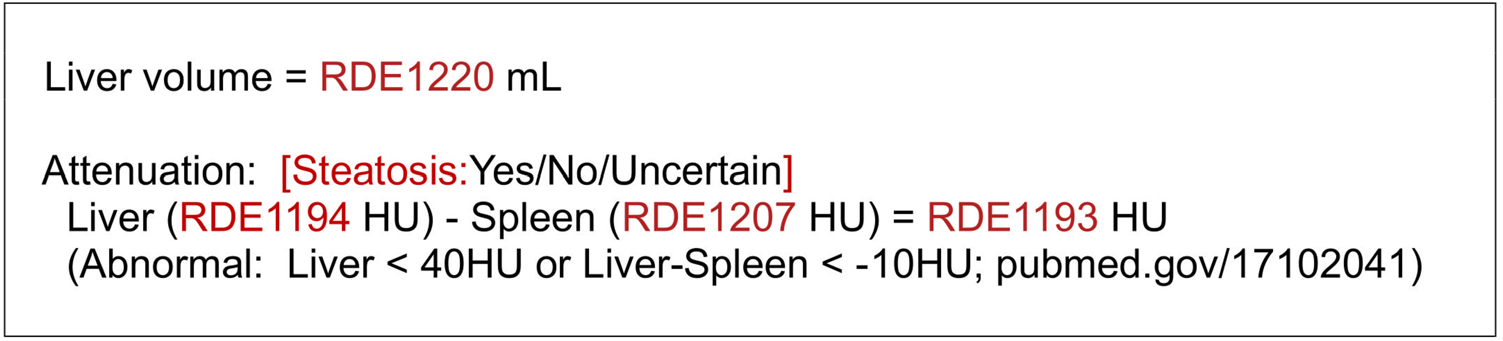
Reporting template for PowerScribe 360 (Microsoft Nuance, Burlington, MA) that allows liver volume and attenuation values to be imported directly into the report from the AI system. Text in red indicates fields filled automatically from AI-derived results (e.g., RDE1220) or entered manually from a “pick list” of pre-defined values (e.g., steatosis)

In our implementation, the AInSights platform generated various image series, such as image overlays, and transmitted them to the institution’s PACS (IDS7, Sectra AB, Linköping, Sweden). The software also generated Digital Imaging and Communications in Medicine Structured Reporting (DICOM-SR) objects that incorporated the CDE labels and values as attribute-value pairs. Commercial software (Laurel Bridge Software, Inc., Newark, DE), already in use at the authors’ institution, extracted the AI-generated DICOM-SR data and inserted the values into template-based reports of the radiology reporting system (PowerScribe 360, Microsoft Nuance, Burlington, MA). The reporting templates used the corresponding CDE identifiers, such as “RDE1193,” as the field labels. No special modifications were required. The reporting template for liver attenuation measurement is shown in Fig. 2.

## Results

**Fig. 3:**
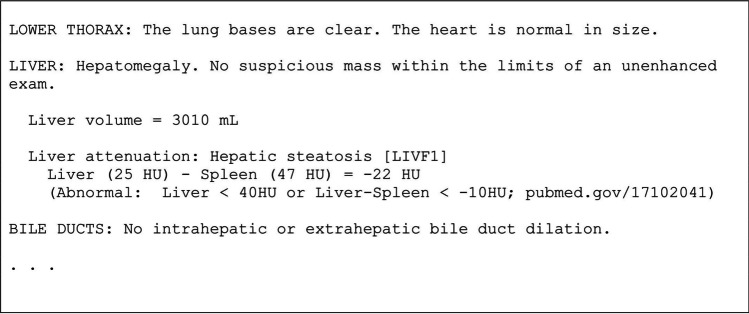
Portion of a radiology report with content incorporated from the AI model. The liver volume and liver and spleen attenuation values are imported directly into the radiology report. The bracketed alphanumeric codes (e.g., “[LIVF1]”) allow one to search the report text for cases of hepatic steatosis

**Fig. 4:**
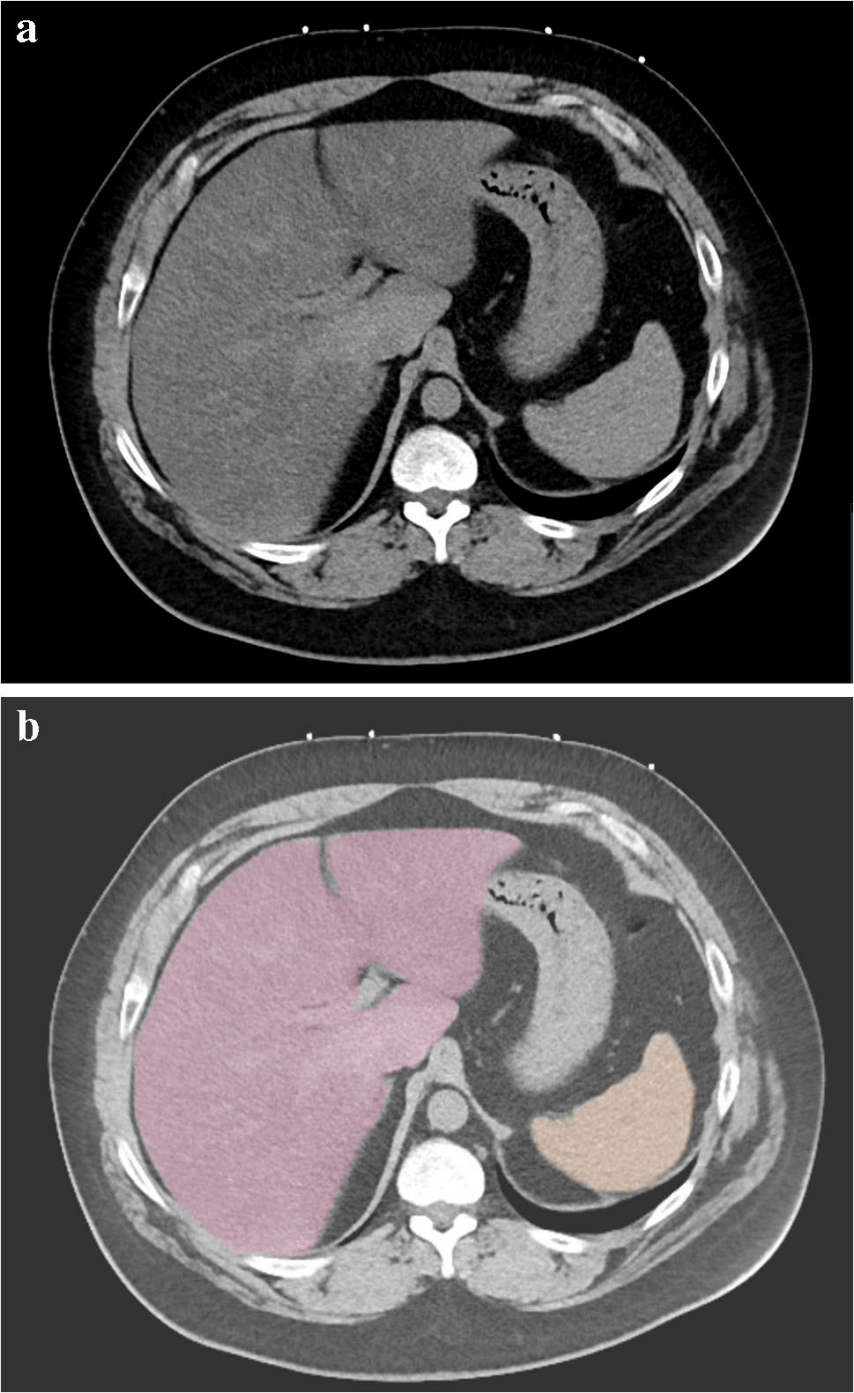
**A** Abdominal CT image of patient with hepatic steatosis. AI analysis yielded a liver volume of 3010 mL with a mean attenuation of 25 HU; mean attenuation of the spleen was 47 HU. **B** Corresponding AI-processed image includes color overlays to highlight the segmentation of the liver (pink) and spleen (yellow)

The AI model successfully segmented the liver and spleen in non-contrast CT images and generated measurements of the organs’ volume and attenuation. Values were incorporated successfully into radiology reports (Fig. 3). In accordance with the AIR profile, AInSights generated a new imaging exam series with color overlays to indicate the AI system’s segmentation of the liver, spleen, visceral fat, subcutaneous fat, and other tissues (Fig. 4). AI-generated image series and associated DICOM-SR data were transmitted to the PACS for storage and display. From May 2023 through February 2024, the AI system analyzed 3920 non-contrast CT examinations, of which 339 exams (8.7%) were positive for hepatic steatosis.

## Discussion

The work presented here underscores the significance of integrating interoperability standards with advanced computational technologies to foster a data-driven culture in radiology. The integration approach supports the care of individual patients and enables the aggregation of data for research and development of radiology AI applications. This manuscript delineates the methodology, outcomes, and insights gleaned from the use of CDEs in clinical practice to record and transfer AI-generated data.

The integration of CDEs in radiology reporting entails several advantages. First and foremost, it promotes interoperability among diverse healthcare systems to foster seamless communication and collaboration among professionals by providing a common language for describing imaging findings. Furthermore, CDEs enhance data quality by standardizing terminology, facilitating data aggregation from multiple sources for research purposes, and enabling robust analysis of large datasets, while simultaneously streamlining the incorporation of new information into electronic health records (EHRs). Workflows involving the integration of AI results into radiology results using DICOM-SR elements have been shown to be both successful and efficient [1], and with CDEs providing a foundation for structured reporting, integrating AI model measurements becomes even more feasible. Jorg et al. developed a pipeline that enables automated pre-population of structured reports with results provided by AI tools [1]. Structured report information enables data mining for quality assurance, epidemiological analysis, and radiology research [14]. CDEs promote language consistency and interoperability of AI-reported findings, assuring that different AI platforms and toolsets speak a common language when referring to specific imaging-derived traits. CDEs are intended for global use: the definitions allow international exchange of data. Automated incorporation of CDE values into radiology reports speeds the reporting process, eliminates the possibility of error in transcribing the values from the AI system to the report, and reduces the radiologist’s cognitive burden.

As artificial intelligence (AI) systems for image analysis have demonstrated increasingly powerful performance, they play an increasing role in clinical medicine in domains such as radiology. It is critical that the data generated by such systems can be integrated readily with imaging reports, with imaging data, and with the EHR. The use of CDEs in AI-based clinical workflows allows for this data integration. The utilization of CDEs in radiology reporting not only optimizes clinical workflows but also opens new avenues for data-driven research, quality improvement initiatives, and benchmarking of diagnostic practices. Furthermore, by enabling objective and consistent comparison of prior and current imaging findings, CDEs can help radiologists provide accurate analysis of disease progression and treatment response to make more informed decisions about patient care. CDE results could be used as input for clinical decision support: for example, AI-generated opportunistic screening values for bone mineral density could be used to formulate follow-up imaging and treatment recommendations.

Implementing this approach requires careful consideration of several key factors, including selection of relevant CDEs, alignment with existing reporting standards, collaboration among stakeholders within the field of radiology (including clinicians, researchers, and informaticians), and ongoing evaluation to refine the system as AI technology evolves. Despite the recognized importance of CDEs in various medical domains, they remain underutilized to incorporate AI-generated radiology data into radiology reports. This study represents an effort to bridge this gap by employing CDEs to systematically record and transfer information produced by an AI system into radiology reports. By doing so, we aim to establish a replicable framework that not only enhances the clarity and utility of AI findings for radiologists but also contributes to cumulative knowledge building through consistent data aggregation.

If CDEs are not adopted when implementing AI-based systems, the resulting output may be ambiguous or inconsistent. For example, systems that measure the diameter of the abdominal aorta to screen for abdominal aortic aneurysm might output a numerical value, but it might not be clear if the diameter is measured in millimeters or centimeters. Does the value represent the maximal anteroposterior diameter (i.e., in the sagittal plane), or the diameter measured in a plane perpendicular to the axis of the vessel? Does it measure the intimal diameter, or is it the external diameter? Without agreed-upon definitions, measurement across different systems cannot be integrated.

Beyond its immediate clinical applications, the creation of a standardized CDE-based framework for processing and analysis of radiology imaging by AI models will enable novel observational health research projects. A primary application is with the Observational Health Data Sciences and Informatics (OHDSI) distributed data network, the largest of its kind in the world [15, 16]. A key characteristic of OHDSI is its standardization of data into the Observational Medical Outcomes Partnership (OMOP) data model, which allows for a vast range of observational projects to be conducted on available data [17]. In conjunction with OMOP, the Radiology Common Data Model (R-CDM) will be used to standardize DICOM metadata for medical imaging files [18]. Storing relevant data features using CDEs will ensure that analyses produced by AI models on clinical data will be able to be efficiently integrated with OHDSI and other relevant data networks, making such data easily accessible for use in research alongside patient care.

In accordance with the Findable, Accessible, Interoperable, and Reusable (FAIR) principles of the National Institutes of Health [19], imaging data analyzed by AI models will be stored in DICOM format using the medical imaging data structure, and non-imaging data both analyzed and produced by AI models will be stored using the HL7 Fast Healthcare Interoperability Resources (FHIR) [20]. These FAIR principles, originally created to address scientific data management, are beginning to be applied formally to AI-based work to promote the sharing of knowledge, enabling new discoveries and innovations that benefit the scientific community as a whole [21]. Additionally, given that FHIR is the industry standard for the secure and efficient transfer of healthcare data across systems, it is essential to verify that CDEs part of the AI framework are fully compatible with FHIR, ensuring that data collected and stored using CDEs can be extracted and transferred as necessary. A framework to standardize imaging findings as FHIR observations using CDEs has been developed successfully [22, 23].

With the standardization of this workflow comes its applicability to fields beyond just radiology; most notably, oncology [24] and radiation oncology [25], for which there are already existing efforts to develop relevant CDEs. Although individual CDEs will most likely differ depending on the specific clinical application, the underlying framework of the process by which findings are extracted and stored from data remains constant, ensuring interoperability across medical fields. Given this inherent generalizability and applicability, there are a few specific limitations of the CDE-based framework. However, given that the investigators focused on evaluating success based on a specific clinical application of liver/spleen abdominal CT segmentation, it may be worthwhile to conduct future proof-of-concept tests with other imaging modalities and anatomic regions to validate the findings laid out in this article. As AI software grows more versatile and becomes more commonplace for the efficient analysis of clinical radiological data collected from patients, the use of CDEs to embed the output of AI models will ensure the standardization, applicability, and efficiency of this process.

## Abbreviations

AI: Artificial intelligence
AIR: AI results
AIW-I: AI Workflow for Imaging
CDE: Common data element
DICOM: Digital Imaging and Communications in Medicine
DICOM-SR: DICOM Structured Reporting
EHR: Electronic health record
FAIR: Findable, Accessible, Interoperable, and Reusable
FHIR: Fast Healthcare Interoperability Resources
IHE: Integrating the Healthcare Enterprise
OHDSI: Observational Health Data Sciences and Informatics
OMOP: Observational Medical Outcomes Partnership
PACS: Picture Archiving and Communication System
R-CDM: Radiology Common Data Model
